# Effect of Maternal Administration with All-Trans Retinoic Acid on Lungs of Neonatal Pigs

**DOI:** 10.3390/vetsci12121132

**Published:** 2025-11-28

**Authors:** Xianghao Xiao, Haimei Zhou, Dehai He, Panting Wei, Yuting Zhu, Wenchen Sun, Shaobin Hao, Huadong Wu, Wei Lu, Yuyong He

**Affiliations:** 1 Jiangxi Province Key Laboratory of Animal Nutrition and Feed, Engineering Research Center of Feed Development, Jiangxi Agricultural University, Nanchang 330045, China; xiaoxianghao10@gmail.com (X.X.); 18679954991@163.com (P.W.); sun1221_wc@163.com (W.S.); haoshaobin1775@163.com (S.H.); lw20030508@163.com (W.L.); 2 College of Animal Science and Technology, Jiangxi Agricultural University, Nanchang 330045, China; whd0618@163.com; 3 Department of Animal Science, Jiangxi Agricultural Engineering Vocational College, Zhangshu 331200, China; 18270826482@163.com; 4 Department of Neurology, Ganzhou People’s Hospital, Ganzhou 341000, China; hedehai1989@163.com; 5 Nanchang Agricultural Technology Extension Center, Nanchang 330009, China; 13870664622@163.com

**Keywords:** neonatal pigs, maternal administration, all-trans retinoic acid, fetal lung, histomorphology, bacterial composition, gene expression

## Abstract

(1) Background: How to reduce respiratory diseases in animals is a matter of great concern, and this study aimed to investigate the effect of maternal administration with all-trans retinoic acid (ATRA) on the lung health of neonatal pigs. (2) Methods: Fifteen sows were randomly allotted to one of five ATRA groups (0, 4, 8, 16, and 32 mg/kg diet), respectively; on average, from days 12 to 95 after insemination, lung samples of neonatal pigs were harvested for HE staining, 16S DNA sequencing, and transcriptomic analysis. (3) Results: Compared to neonatal pigs from the ATRA0 group, neonatal pigs from the ATRA4 group had an increased (*p* > 0.05) alveolar number and the thinner (*p* < 0.05) alveolar septum, higher (*p* < 0.05) abundance of *Akkermansia* and *unidentified_Mitochondria*, and a lower (*p* < 0.05) abundance of *Acinetobacter*, *Cutibacterium*, *Stenotrophomonas*, *Enterobacter*, *Saccharomonospora*, and *Alistipes* in the lungs. Maternal supplementation of ATRA at 4 mg/kg diet had the benefits of reducing pathogen virulence and drug resistance, but it poses the risk of increasing the resistance to β-lactam antibiotics. In addition, it reduced (*p* < 0.05) the virulence, drug resistance, and stress tolerant capacity of potential pathogens (Proteobacteria, *Acinetobacter*, *Cupriavidus*, and *Pseudomonas*), enriched the highest number of genes in neuroactive ligand–receptor interaction pathway, and decreased gene expression of CCL11 and IL9 in the asthma pathway. (4) Conclusions: Maternal supplementation of ATRA at 4 mg/kg diet can strengthen the lung health of neonatal pigs by improving alveolar development, decreasing the number and virulence of pathogens, and down-regulating the expression of asthma-related genes.

## Simple Summary

Reducing animal respiratory diseases has received widespread attention as it can effectively improve animal health, productivity, and economic outcomes. This study investigated the effect of maternal administration with all-trans retinoic acid (ATRA) at different levels on the lung health of neonatal pigs; results indicated that maternal supplementation of ATRA at 4 mg/kg diet from days 12 to 95 after insemination was optimal for the lung health of neonatal pigs, because this practice can strengthen the lung health of neonatal pigs by improving alveolar development, decreasing the number and virulence of pathogens, and down-regulating the expression of asthma-related genes. These findings contribute to the development of strategies for controlling respiratory disease in postnatal animals.

## 1. Introduction

The healthy development of fetal lungs is vital for postnatal outcomes; disruptions in this process can lead to malformation and dysfunction of fetal lungs, which are directly linked to lots of postnatal lung diseases including asthma and respiratory disease complex [1,2,3,4]. Fetal lung development is a complex process; it is influenced by genetics, maternal health, pregnant status, and nutrition [2,4,5,6]. Previous studies reported that Hoxa1 mutation resulted in congestion and edema in the lungs of neonatal pigs [4]; chronic exposure to chorioamnionitis induced lung injury and inflammation and increased surfactant protein expression [7].

Nutrition is one of the most important factors in fetal lung development, because a proper supply of vitamins [8], minerals [9], energy, and proteins [10] is necessary for healthy lung growth. During the alveolar stage of lung development, the lack of nutrients can damage the expression of surfactant proteins in fetal lungs in sheep, restrict pulmonary vascular growth, and reduce the alveolar surface area [11]. The lack of vitamin D and vitamin C can lead to functional and structural disorders of the lungs [8,12]; vitamin A deficiency in maternal food may lead to the development of defective lungs, compromise lung function during postnatal life [13,14], and exacerbate allergic asthma [15]. Retinoic acid (RA) is a metabolite of vitamin A; it can ameliorate septation abnormalities [16] and adjust the tight junction permeability of the alveolar epithelium in response to air–liquid interface conditions [17]. RA also can influence the proximal–distal patterning and the branching morphogenesis of lungs by redirecting glucose towards the production of pyruvate and succinate [18]. RA treatment can alleviate allergy symptoms and airway inflammation [19,20]. RA administration during pregnancy can reverse lung malformation with the effects on the radial alveolar count, type II/type I ratio, and surfactant protein expression [21].

ATRA (all-trans retinoic acid) is also an active metabolite of vitamin A; it is often used as a potent modulator for lung development [16,22], and an excess or deficiency of ATRA has serious effects on embryonic development, including fetal lungs [23,24]. ATRA may mitigate airway remodeling associated with asthma by enhancing RAR signaling [15]. Chen et al. (2023) reported that Hoxa1 mutation in neonatal pigs developed into dyspnea characterized by congestion and edema in the lungs, and supplementation of ATRA to crossbred sows at 4 mg/kg BW on dpc 14 rescued lung malformation in Hoxa1 mutant neonatal pigs by increasing airspace area and microvascular density and decreasing septal thickness and surfactant protein C (SFTPC) expression [4]. In addition, a report showed that ATRA can inhibit fungal growth and prevent the production of biofilms by pathogens [25].

Fetal lung malformations contribute significantly to postnatal morbidity and mortality in animals [26]. In this study, crossbred sows were used as a model to investigate the impact of maternal supplementation with varying ATRA levels on fetal lung development, focusing on three key aspects: inflammatory response, developmental trajectory, and bacterial community composition. This study aims to identify an optimal ATRA dosage that promotes fetal lung health and reduces susceptibility to postnatal lung diseases.

## 2. Materials and Methods

### 2.1. Animal Feeding and Sample Harvesting

This experiment was conducted on a commercial pig farm; after artificial insemination, all sows were housed individually in slatted gestation pens and then transferred into farrowing pens on gestation day 107 and housed in identical individual stalls on a slatted floor. Fifteen crossbred sows (Landrace × Large white), artificially inseminated with semen collected from healthy Duroc boar, were randomly assigned to one of five groups treated with all-trans retinoic acid (ATRA, Sigma (Sigma-Aldrich(Shanghai)Trading Co., Ltd., Shanghai, China), purity ≥ 98.0%): ATRA0 (supplementation of ATRA at 0 mg/kg basal diet), ATRA4 (supplementation of ATRA at 4 mg/kg basal diet), ATRA8 (supplementation of ATRA at 8 mg/kg basal diet), ATRA16 (supplementation of ATRA at 16 mg/kg basal diet), and ATRA32 (supplementation of ATRA at 32 mg/kg basal diet). Each group had 3 replicates, and each replicate had 1 pregnant sow. From the 12th to 95th day after insemination, sows in each group were offered the basal diet (60% corn, 14% soybean meal, 13% beet pulp, 9% wheat bran, and 4% premix) containing ATRA mixture at 7:30 and 17:00, respectively, and prior to feeding, half of the dietary allowance was blended evenly with the ATRA mixture (ATRA was firstly dissolved in dimethyl sulfoxide at 1:62.5 and then diluted with soybean oil at 1:1) [27]. Sows in five treatment groups were offered the basal diet without ATRA addition from the 96th day of pregnancy to farrowing day.

Prior to suckling, two newborn piglets (1♂, 1♀) closest to the average bodyweight were selected from each litter and then euthanized with pentobarbital sodium (100 mg/kg body weight) according to the protocol approved by the Animal Ethics Committee of Jiangxi Agricultural University (JXAULL-2024-10-01). After opening the chest cavity, lung samples were harvested from the same right lobe location, and each ATRA treatment group had 6 newborn pigs (2 piglets/sow × 3 sows/ATRA treatment group) for lung sampling. Lung samples for histomorphological staining were fixed in 4% paraformaldehyde solution, and samples for other analysis were flash-frozen in liquid nitrogen and then stored in an ultra-low temperature freezer at −80 °C for later processing.

### 2.2. Enzyme-Linked Immunosorbent Assay (ELISA) of Cytokines

The level of interleukin 1β (IL-1β), IL-6, IL-10, IL-13, and tumor necrosis factor-α (TNF-α) in lung samples were measured using commercial ELISA kits (Shanghai Yuan Orange Biotechnology Center, Shanghai, China) according to the protocols of the manufacturer.

### 2.3. Histomorphological Measurement

First, lung samples were fixed in 4% paraformaldehyde solution for 24 h, then embedded with paraffin wax, sliced into sections with a thickness of 5 µm, and stained with hematoxylin and eosin (HE). Image-Pro Plus 6.0 software (Media Cybernetics, Inc., Bethesda, MD, USA) was used to measure the number and septal wall thickness of alveoli of sections under 200× magnification [28].

### 2.4. Immunofluorescence Analysis

After dewaxing, antigen-repairing, and blocking with 10% normal donkey serum, sections were incubated with antibody AQP5 (Starter, S0B1287) or SFTPC (Starter, S0B1215) overnight in a refrigerator at 4 °C. Following incubation, slides were taken out and washed with PBS (phosphate-buffered saline, pH 7.4). Again, slides were incubated with the secondary antibodies and washed with PBS and finally stained with DAPI (4′,6-diamidino-2-phenylindole, Solarbio, C0060). The Pannoramic MIDI scanner (3D HISTECH Ltd., Budapest, Hungary) and Case Viewer software, 2.4.0 version, (3D-HISTECH Ltd., Budapest, Hungary) were used to scan slides and to capture target images, respectively.

### 2.5. Bacterial Community Composition Analysis

The bacterial DNA of neonatal pig lungs was extracted using the cetyl trimethyl ammonium bromide (CTAB) method [29] and then stored in a refrigerator at −20 °C, followed by measuring the concentration of extracted DNA using a NanoDrop 1000 spectrophotometer (Thermo Fisher Scientific, Karlsruhe, Germany). The template DNA of lung tissues was amplified using primers (341F: 5′-CCTAYGGGRBGCASCAG-3′, 806R: 5′-GGACTACNNGGGTATCTAAT-3′) by targeting the 16S V3 region and V4 region in 30 µL reaction (15 µL of Phusion ➅ High-Fidelity PCR Master Mix, 0.2 µL of Primer F, 0.2 µL of Primer R and 10 µL of g DNA) under the following conditions: 98 °C for 1 min, followed by 30 cycles of 98 °C for 10 s, 50 °C for 30 s, 72 °C for 30 s, and a final extension of 72 °C for 5 min [30].

Amplicon libraries were generated with the purified PCR products after quantification using a Qubit 3.0 Fluorometer (Invitrogen, Waltham, MA, USA); then, the libraries were pooled in equimolar amounts and paired-end-sequenced (PE250) at Novogene Co., Ltd. (Beijing, China) on an Illumina NovaSeq 6000 platform (Illumina, San Diego, CA, USA). Bioinformatics analysis was conducted according to a previously published method [30]. Briefly, the MetaStat analysis was carried out with R software (V3.5.3) to find out the significantly different species at phylum and genus level, respectively. The linear discriminant analysis (LDA) effect size (LEfSe) analysis was performed to find out the biomarkers. Phylogenetic Investigation of Communities by Reconstruction of Unobserved States 2 (PICRUSt2) software was used to study the function of the bacterial community, and the bacterial functions were annotated using the Clusters of Orthologous Groups of Proteins (COGs) database (https://www.ncbi.nlm.nih.gov/COG/, accessed on 15 September 2025). The bacterial phenotypes were predicted by searching the BugBase database (https://bugbase.cs.umn.edu/, accessed on 15 September 2025). Raw data of 16S rDNA sequencing have been submitted to the database of Sequence Read Archive of the National Center for Biotechnology Information with the BioProject ID PRJNA1233939.

### 2.6. RNA Sequencing and Data Analysis

The total RNA of lung samples was extracted with TRNzol Universal Reagent (TianGen Biotechnology, Beijing, China) according to the manufacturer’s instructions. The integrity and purity of RNA were measured using the Agilent 5400 Bioanalyzer (Agilent Technologies, Palo Alto, CA, USA). The Fast RNA-seq Lib Prep Kit V2 (ABclonal, Wuhan, China) was used to construct the RNA-Seq libraries. After quality inspection through the touch q-PCR system CFX96 (BIO-RAD, Hercules, CA, USA), the qualified libraries were sequenced on the NovaSeq 6000 platform (Illumina, San Diego, CA, USA). The fastp (version 0.19.7) was used to filter and trim raw reads, and clean reads were aligned to the reference genome using HISAT2. Fragments Per Kilobase of transcript per Million mapped fragments (FPKM) with Cufflinks were used to calculate the expression levels of each gene, and the read count of each gene were generated by HTSeq. DESeq 2 was used to identify the differentially expressed genes (DEGs) with *p*-value < 0.05 and |log2 FC| > 2. KEGG pathway enrichment analysis was carried out to explore the functions of DEGs [29].

### 2.7. Statistical Analysis

Statistical analyses were performed using the R package (version 3.6.1; R Foundation for Statistical Computing, Vienna, Austria). The ordinary one-way analysis of variance (ANOVA) followed by Bonferroni post hoc tests were performed for comparing cytokine levels and histomorphological data. The non-parametric Wilcoxon test or the non-parametric Kruskal–Wallis rank-sum test was employed in the analysis of the lung microbiota. Data are expressed as the mean and standard error of the mean (SEM).

## 3. Results

### 3.1. Comparison of the Levels of Cytokines in the Lungs of Neonatal Pigs in Different Groups

Data in Table 1 showed that neonatal pigs in the ATRA16 group had higher levels of IL-1β (*p* < 0.05), IL-6 (*p* < 0.01), IL-10 (*p* < 0.05), IL-13 (*p* < 0.05), and TNF-α (*p* < 0.05) in the lungs than neonatal pigs in the ATRA32 group, respectively. In addition, neonatal pigs from the ATRA8 group had lower IL-6 (*p* < 0.01), IL-13 (*p* < 0.05), and TNF-α (*p* < 0.05) levels in the lungs than pigs from ATRA16, but lower IL-6 (*p* < 0.01) levels in the lungs than pigs from the ATRA0 group. No significant differences (*p* > 0.05) were found in the levels of IL-1β, IL-6, IL-10, IL-13, and TNF-α of lung between the ATRA0 group and ATRA8 group.

### 3.2. Histomorphological Alteration in Lungs of Neonatal Pigs from Different Groups

**Figure 1:**
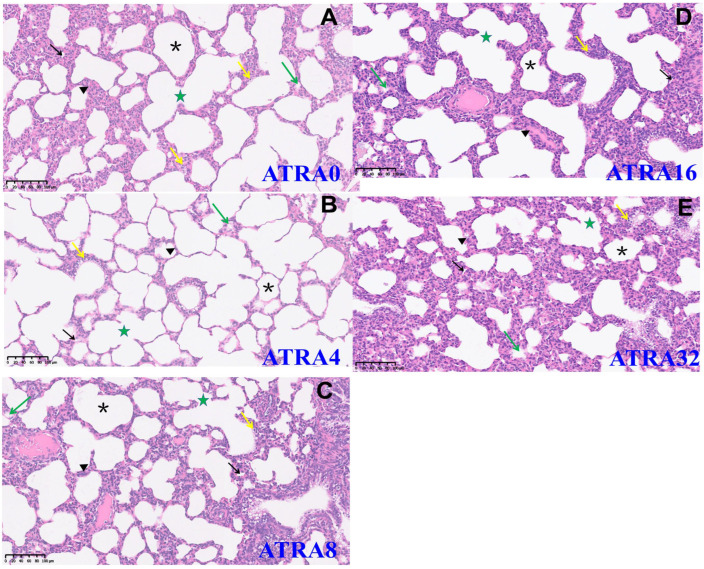
HE staining images of lungs of neonatal pigs from different ATRA treatment groups (*n* = 6 per group). ATRA0: Supplementation of ATRA at 0 mg/kg basal diet; ATRA4: supplementation of ATRA at 4 mg/kg basal diet; ATRA8: supplementation of ATRA at 8 mg/kg basal diet; ATRA16: supplementation of ATRA at 16 mg/kg basal diet; ATRA32: supplementation of ATRA at 32 mg/kg basal diet. Alveoli: asterisk; alveolar septum: triangle; intra-alveolar edema: green arrow; alveolar fusion: pentagram; inflammatory cell infiltration: yellow arrow; Alveolar consolidation: black arrow. Magnification: 200×. (**A**), HE staining images of lungs of neonatal pigs from ATRA0 group. (**B**), HE staining images of lungs of neonatal pigs from ATRA4 group. (**C**), HE staining images of lungs of neonatal pigs from ATRA8 group. (**D**), HE staining images of lungs of neonatal pigs from ATRA16 group. (**E**), HE staining images of lungs of neonatal pigs from ATRA32 group.

**Figure 2:**
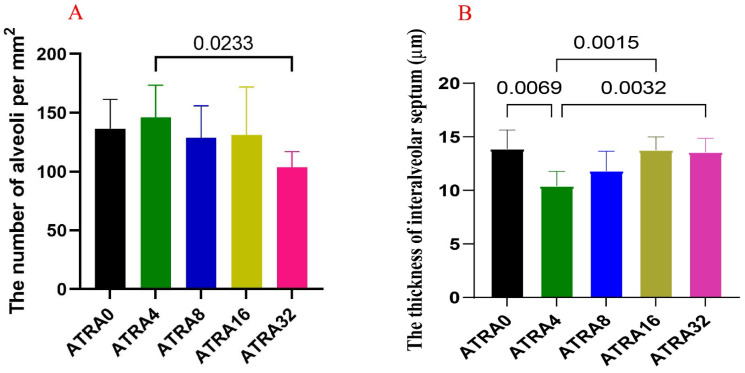
Effects of maternal supplementation with different ATRA levels on the number and septum thickness of alveoli (*n* = 6 per group). ATRA0: supplementation of ATRA at 0 mg/kg basal diet; ATRA4: supplementation of ATRA at 4 mg/kg basal diet; ATRA8: supplementation of ATRA at 8 mg/kg basal diet; ATRA16: supplementation of ATRA at 16 mg/kg basal diet; ATRA32: supplementation of ATRA at 32 mg/kg basal diet. (**A**) Comparison of alveolar numbers. (**B**) Comparison of alveolar septum thickness. Data are presented as mean ± SE.

HE staining (Figure 1) showed that symptoms of alveolar collapse (consolidation), inflammatory cell infiltration, and alveolar fusion were found in five ATRA treatment groups; however, the ATRA4 group had less alveolar collapse (consolidation) and slight intra-alveolar edema. Figure 2 indicates that neonatal pigs from the ATRA4 group had the highest number of alveoli among the five ATRA treatment groups, but only neonatal piglets from the ATRA4 group had an increased (*p* < 0.05) number of alveoli than piglets from the ATRA32 group. In addition, neonatal piglets from the ATRA4 group had a thinner (*p* < 0.01) alveolar septum than neonatal piglets from the ATRA0, ATRA16, and ATRA32 groups, respectively.

### 3.3. Alveolar Expression of AQP5 and SFTPC

**Figure 3:**
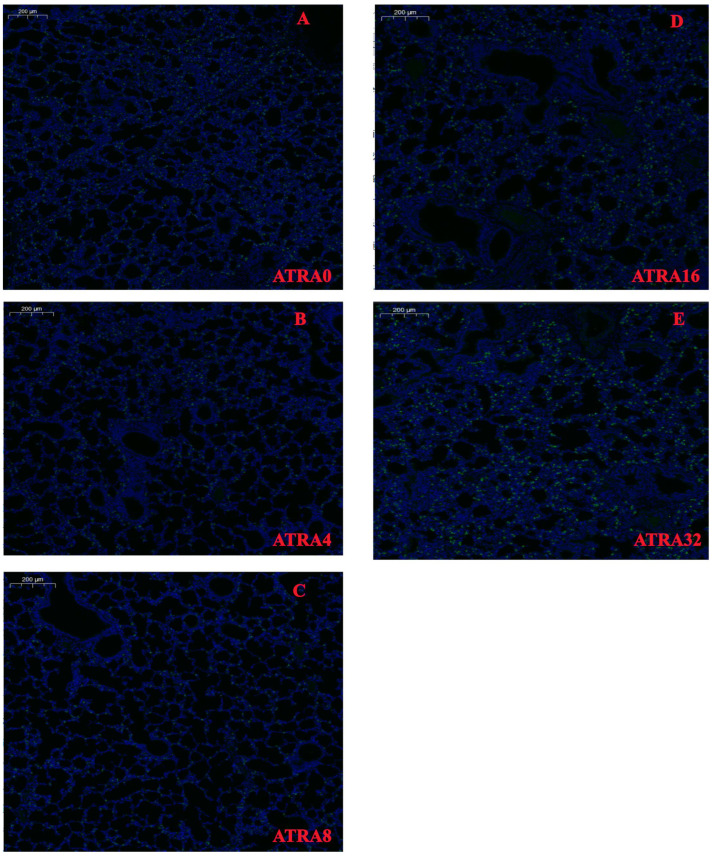
SFTPC distribution with immunofluorescence staining for lung samples in different ATRA treatment groups (*n* = 6 per group). ATRA0: supplementation of ATRA at 0 mg/kg basal diet; ATRA4: supplementation of ATRA at 4 mg/kg basal diet; ATRA8: supplementation of ATRA at 8 mg/kg basal diet; ATRA16: supplementation of ATRA at 16 mg/kg basal diet; ATRA32: supplementation of ATRA at 32 mg/kg basal diet. (**A**–**E**) Image of SFTPC expression colored with green (SFTPC) and blue (DPAI).

**Figure 4:**
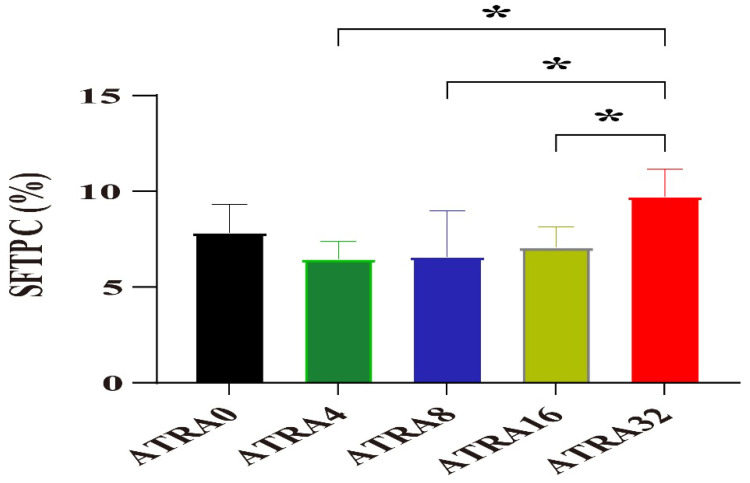
Effects of maternal supplementation with different ATRA levels on the relative expression of SFTPC in alveoli (*n* = 6 per group). ATRA0: supplementation of ATRA at 0 mg/kg basal diet; ATRA4: supplementation of ATRA at 4 mg/kg basal diet; ATRA8: supplementation of ATRA at 8 mg/kg basal diet; ATRA16: supplementation of ATRA at 16 mg/kg basal diet; ATRA32: supplementation of ATRA at 32 mg/kg basal diet. Data are presented as mean ± SE, and *, *p* < 0.05.

**Figure 5:**
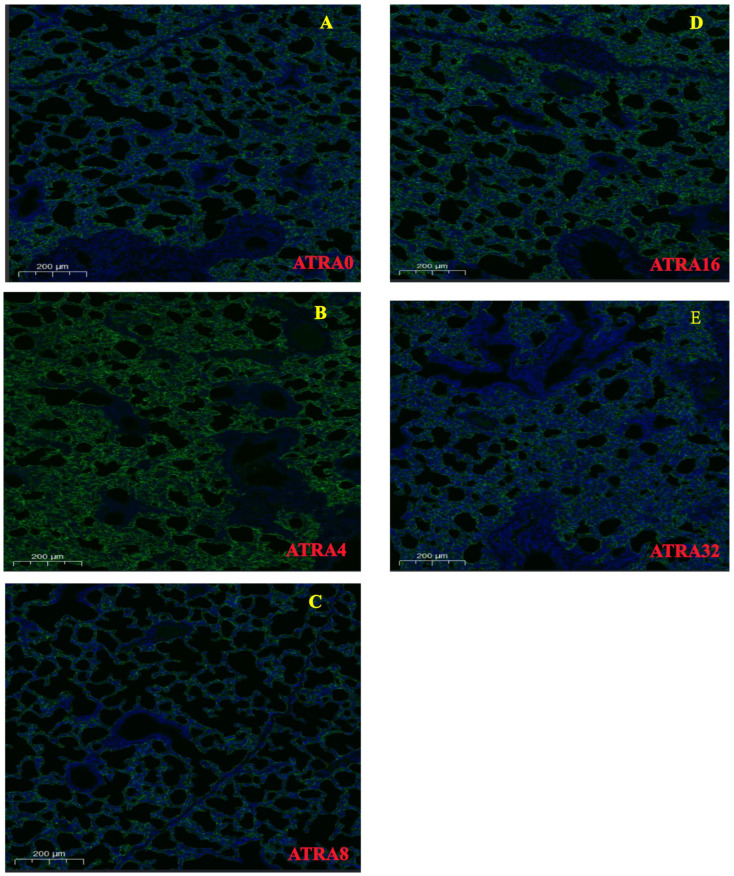
AQP5 distribution with immunofluorescence staining for lung samples in different ATRA treatment groups (*n* = 6 per group). ATRA0: supplementation of ATRA at 0 mg/kg basal diet; ATRA4: supplementation of ATRA at 4 mg/kg basal diet; ATRA8: supplementation of ATRA at 8 mg/kg basal diet; ATRA16: supplementation of ATRA at 16 mg/kg basal diet; ATRA32: supplementation of ATRA at 32 mg/kg basal diet. (**A**–**E**) Images of SFTPC expression colored with green (SFTPC) and blue (DPAI). (**A**–**E**) Images of AQP5 expression colored with green (AQP5) and blue (DPAI).

**Figure 6:**
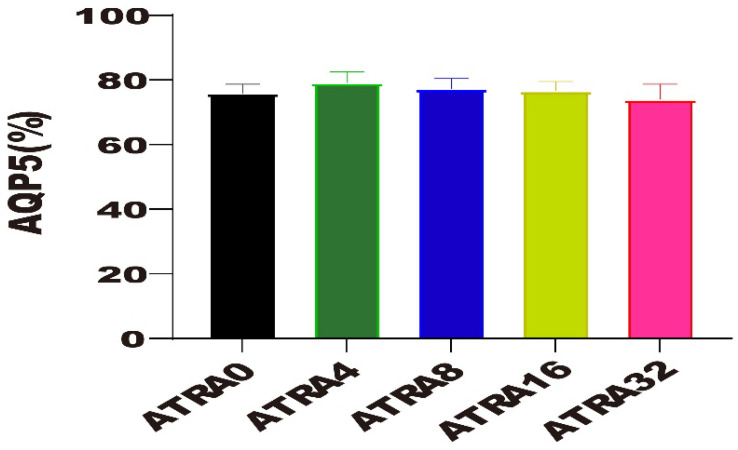
Effects of maternal supplementation with different ATRA levels on the relative expression of AQP5 in alveoli (*n* = 6 per group). ATRA0: supplementation of ATRA at 0 mg/kg basal diet; ATRA4: supplementation of ATRA at 4 mg/kg basal diet; ATRA8: supplementation of ATRA at 8 mg/kg basal diet; ATRA16: supplementation of ATRA at 16 mg/kg basal diet; ATRA32: supplementation of ATRA at 32 mg/kg basal diet. Data are presented as mean ± SE.

The expressions of SFTPC in alveoli of different ATRA treatment groups are presented in Figure 3. Data in Figure 4 show that compared to neonatal pigs from the ATRA32 group, neonatal pigs from the ATRA4, ATRA8, and ATRA16 groups had significant lower (*p* < 0.05) SFTPC expression in the alveoli, respectively. The supplementation of ATRA to pregnant sows numerically decreased the expression of SFTPC in the alveoli of neonatal pigs, with the exception of the diet with ATRA addition at 32 mg/kg. The expressions of AQP5 in alveoli of different ATRA treatment groups are presented in Figure 5. The results in Figure 6 indicated that maternal administration with ATRA had no significant influence (*p* > 0.05) on the expression of AQP5 in alveoli, but supplementation of ATRA to pregnant sows numerically increased the expression of AQP5 in the alveoli of neonatal pigs.

### 3.4. The Diversity and Composition of Lung Bacteria

**Figure 7:**
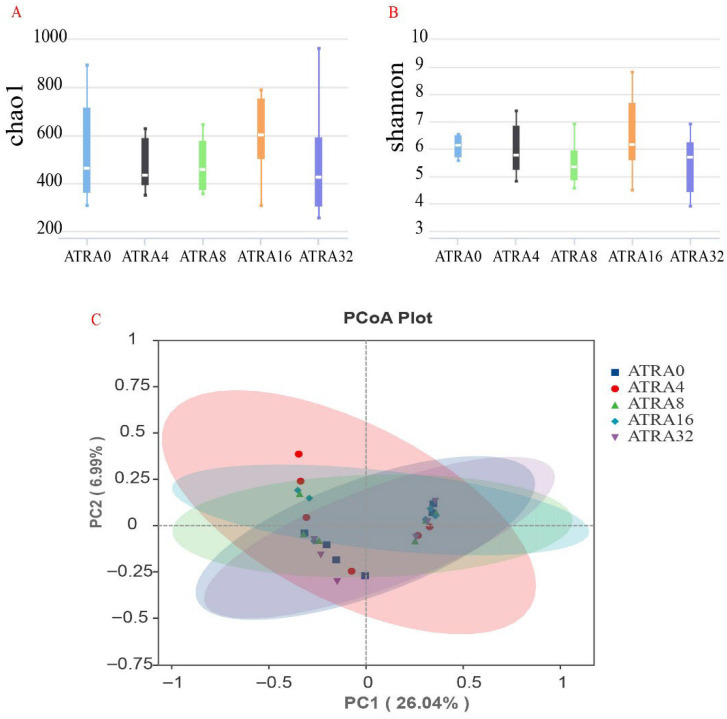
Bacterial diversity in lungs of neonatal pigs in different ATRA treatment groups (*n* = 6 per group). ATRA0: supplementation of ATRA at 0 mg/kg basal diet; ATRA4: supplementation of ATRA at 4 mg/kg basal diet; ATRA8: supplementation of ATRA at 8 mg/kg basal diet; ATRA16: supplementation of ATRA at 16 mg/kg basal diet; ATRA32: supplementation of ATRA at 32 mg/kg basal diet. (**A**) Chao1 index. (**B**) Shannon index. (**C**) PCoA plot.

**Figure 8:**
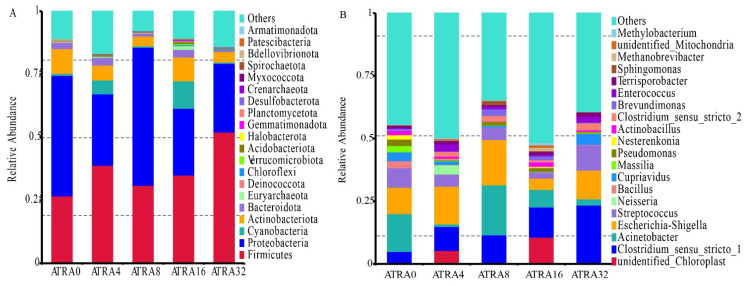
Bacterial composition of lungs of neonatal pigs in different ATRA treatment groups (*n* = 6 per group). ATRA0: supplementation of ATRA at 0 mg/kg basal diet; ATRA4: supplementation of ATRA at 4 mg/kg basal diet; ATRA8: supplementation of ATRA at 8 mg/kg basal diet; ATRA16: supplementation of ATRA at 16 mg/kg basal diet; ATRA32: supplementation of ATRA at 32 mg/kg basal diet. (**A**) Phylum level. (**B**) Genus level.

**Figure 9:**
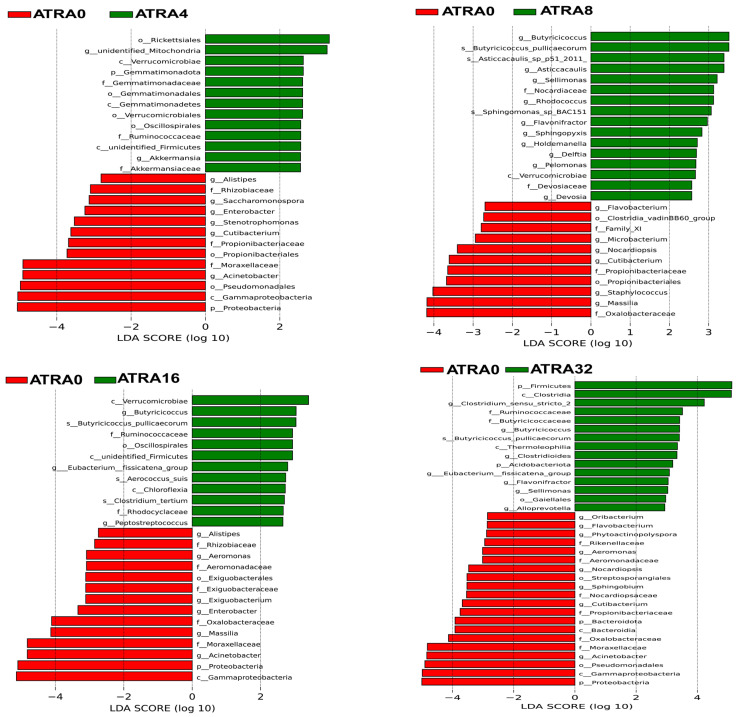
LEfSe analysis of differential bacterial taxa of lungs of neonatal pigs between groups (*n* = 6 per group). ATRA0: supplementation of ATRA at 0 mg/kg basal diet; ATRA4: supplementation of ATRA at 4 mg/kg basal diet; ATRA8: supplementation of ATRA at 8 mg/kg basal diet; ATRA16: supplementation of ATRA at 16 mg/kg basal diet; ATRA32: supplementation of ATRA at 32 mg/kg basal diet. Each plot shows taxa with significant differences in abundance between groups, with the LDA score (log 10) indicating the effect size. LDA score ≥ 2.5.

No significant difference (*p* > 0.05) was found in alpha diversity (Chao1 index, Shannon index) of bacterial communities of lungs when comparing one group to the others (Figure 7A,B). Results for the beta diversity (PCoA analysis) of bacteria showed that there was no significant clustering based on unweighted unifrac distance (Figure 7C). The bacterial composition of lungs is presented in Figure 8, and data showed that the dominant bacteria phylum of lungs shared by neonatal pigs of five treatment groups were Firmicutes, Proteobacteria, and Actinobacteriota (Figure 8A). Supplementation of ATRA in pregnant sows increased the relative abundance of Firmicutes in the lungs of neonatal pigs, and LEfSe analysis (Figure 9) indicated that neonatal pigs in the ATRA0 group had a higher (*p* < 0.05) relative abundance of Proteobacteria in lungs than neonatal pigs in ATRA4, ATRA16, and ATRA32 groups, respectively. Neonatal pigs in the ATRA4 group had a higher (*p* < 0.05) relative abundance of Gemmatimonadota in lungs than neonatal pigs in the ATRA0 group; in addition, neonatal pigs in the ATRA32 group had a higher (*p* < 0.05) relative abundance of Firmicutes and Acidobacteriota in the lungs than neonatal pigs in the ATRA0 group.

The administration of ATRA to pregnant sows also shaped the bacterial composition of the lungs at the genus level in neonatal pigs (Figure 8B). Neonatal pigs in the ATRA0 group and ATRA8 group had the dominant bacteria of *Acinetobacter*, *Escherichis-Shigella*, *Streptococcus*, and *Clostridium_sunsu_stricto_1* in the lungs, neonatal pigs in the ATRA4 group had the dominant bacteria of *Escherichia-Shigella*, *Clostridium_sensu_stricto_1*, *unidentified_Chloroplast*, and *Streptococcus* in the lungs, neonatal pigs in the ATRA16 group had the dominant bacteria of *unidentified_Chloroplast*, *Clostridium_sensu_stricto_1*, *Acinetobacter*, and *Escherichia-Shigella* in the lungs, and finally neonatal pigs in the ATRA32 group had the dominant bacteria of *Clostridium_sensu_stricto_1*, *Escherichia-Shigella*, *Streptococcus*, and *Cupriavidus* in the lungs. LEfSe analysis (Figure 9) showed that compared to neonatal pigs in the ATRA0 group, neonatal pigs in the ATRA4 group had a higher (*p* < 0.05) relative abundance of *unidentified_Mitochondria* and *Akkermansia* but a lower (*p* < 0.05) relative abundance of *Acinetobacter*, *Cutibacterium*, *Stenotrophomonas*, *Enterobacter*, *Saccharomonospora*, and *Alistipes* in the lungs. Neonatal pigs in the ATRA8 group had a higher (*p* < 0.05) relative abundance of *Butyricicoccus*, *Asticcacanlis*, *Sellimonas*, *Rhodococcus*, *Flavonifractor*, *Sphingopyxis*, *Delftia*, *Holdemanella*, *Pelomonas*, and *Devosia* and a lower (*p* < 0.05) relative abundance of *Massilia*, *Staphylococcus*, *Cutibacterium*, *Nocardiopsis*, *Microbacterium*, and *Flabobacterium* in the lungs than neonatal pigs in the ATRA0 group. Lungs of neonatal pigs in the ATRA16 group had a higher (*p* < 0.05) relative abundance of *Butyricicoccus*, *Eubacterium_fissicatena_group*, and *Peptostreptococcus* and lower (*p* < 0.05) relative abundance of *Acinetobacter*, *Massilia*, *Euterobacter*, *Exignobacterium*, *Aeromonas*, and *Alistipes* than lungs of neonatal pigs in the ATRA0 group. Compared to the lungs of neonatal pigs in the ATRA0 group, lungs of neonatal pigs in the ATRA32 group had a higher (*p* < 0.05) relative abundance of *Clostridium_sensu_stricto_2*, *Butyricicoccus*, *Clostridioides*, *Eubacterium_fissicatena_group*, *Sellimonas*, *Flavonifractor*, and *Alloprevotella* and lower (*p* < 0.05) relative abundance of *Acinetobacter*, *Cutibacterium*, *Sphingobium*, *Nocardiopsis*, *Aeromonas*, *Phytoactinopolyspora*, *Flavobacterium*, and *Oribacterium*.

### 3.5. Prediction of Lung Bacterial Functions

**Figure 10:**
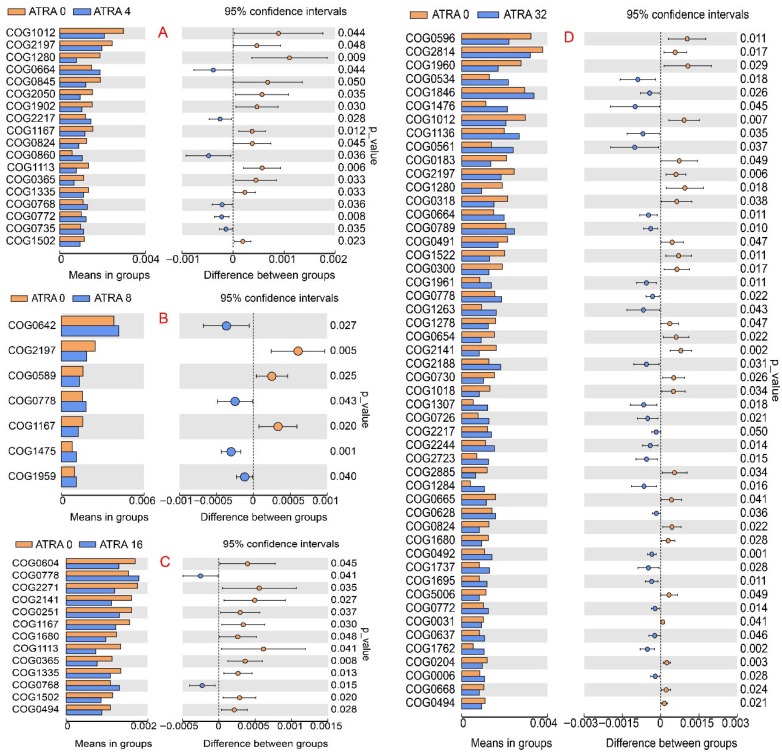
The gene function abundance of bacteria in lungs with statistical difference between groups (*n* = 6 per group). ATRA0: supplementation of ATRA at 0 mg/kg basal diet; ATRA4: supplementation of ATRA at 4 mg/kg basal diet; ATRA8: supplementation of ATRA at 8 mg/kg basal diet; ATRA16: supplementation of ATRA at 16 mg/kg basal diet; ATRA32: supplementation of ATRA at 32 mg/kg basal diet. (**A**), Comparison of significantly altered gene functions between ATRA0 group and ATRA4 group. (**B**), Comparison of significantly altered gene functions between ATRA0 group and ATRA8 group. (**C**), Comparison of significantly altered gene functions between ATRA0 group and ATRA16 group. (**D**), Comparison of significantly altered gene functions between ATRA0 group and ATRA32 group.

The functions of lung bacterial genes were annotated using PICRUSt2 (Phylogenetic Investigation of Communities by Reconstruction of Unobserved States 2) software by blasting bacterial genes against clusters of orthologous groups of proteins (COGs). The results in Figure 10 demonstrated that 18 significantly altered gene functions were found between the ATRA0 group and ATRA4 group, including 12 down-regulated gene functions and 6 up-regulated gene functions; 7 significantly changed gene functions were annotated between the ATRA0 group and ATRA8 group, including 3 down-regulated gene functions and 4 up-regulated gene functions; 13 significantly altered gene functions were identified between the ATRA0 group and ATRA16 group, including 11 down-regulated gene functions and 2 up-regulated gene functions; and 50 significantly altered gene functions were discovered between the ATRA0 group and ATRA32 group, including 25 down-regulated gene functions and 25 up-regulated gene functions. Bacterial functions related to antibiotic resistance, virulence, quorum sensing, drug delivery, and antibiotic biosynthesis included COG2197 (DNA-binding response regulator, NarL/FixJ family, contains REC and HTH domains), COG1280 (Threonine/homoserine/homoserine lactone efflux protein), COG0845 (Multidrug efflux pump subunit AcrA), COG1113 (L-asparagine transporter and related permeases), COG0768 (Cell division protein FtsI/penicillin-binding protein 2), COG0735 (Fe2+ or Zn2+ uptake regulation protein), COG1959 (DNA-binding transcriptional regulator, IscR family), COG1680 (CubicO group peptidase, beta-lactamase class C family), COG2814 (Predicted arabinose efflux permease, MFS family), COG0534 (Na + -driven multidrug efflux pump), COG1846 (DNA-binding transcriptional regulator, MarR family), COG0789 (DNA-binding transcriptional regulator, MerR family), COG1522 (DNA-binding transcriptional regulator, Lrp family), COG2188 (DNA-binding transcriptional regulator, GntR family), COG1307 (Fatty acid-binding protein DegV), COG0492 (Thioredoxin reductase), and COG1695 (DNA-binding transcriptional regulator, PadR family).

### 3.6. Phenotype Analysis of Bacterial Communities

**Figure 11:**
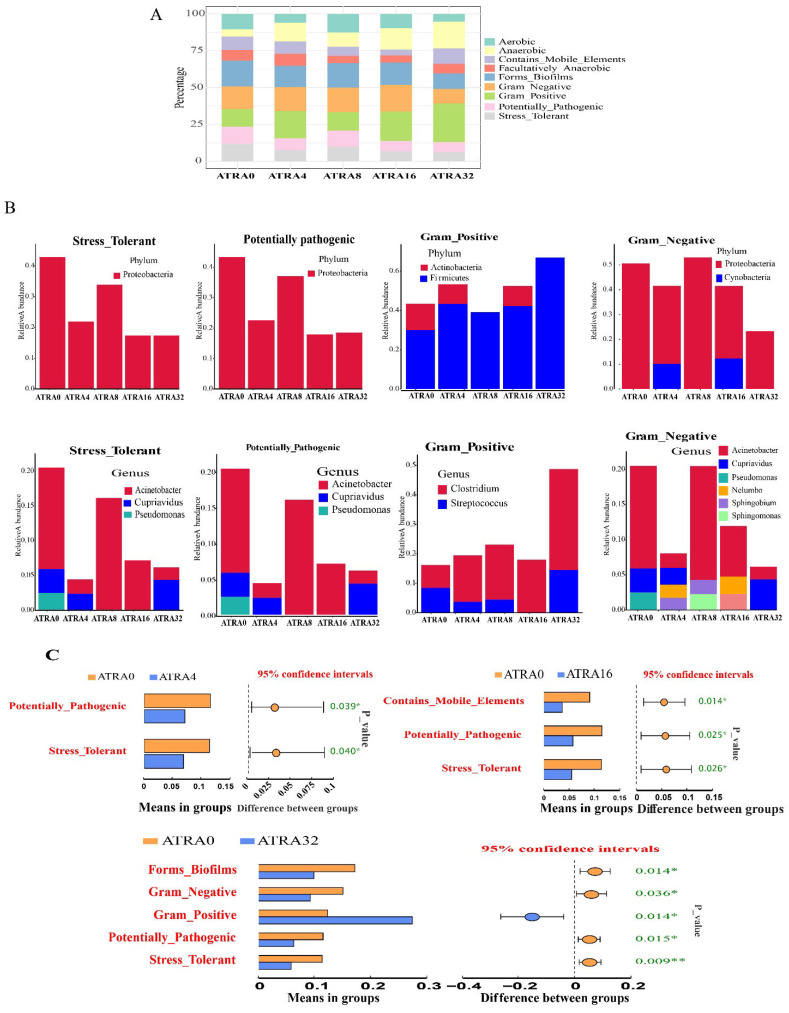
Composition and prediction of BugBase phenotype of bacteria in lungs (*n* = 6 per group). ATRA0: supplementation of ATRA at 0 mg/kg basal diet; ATRA4: supplementation of ATRA at 4 mg/kg basal diet; ATRA8: supplementation of ATRA at 8 mg/kg basal diet; ATRA16: supplementation of ATRA at 16 mg/kg basal diet; ATRA32: supplementation of ATRA at 32 mg/kg basal diet. (**A**) Phenotype composition of bacterial communities. (**B**) Phenotype contribution of bacterial communities. (**C**) Phenotype analysis of bacterial communities. *, *p* < 0.05; **, *p* < 0.01.

The data in Figure 11 show that a total of nine phenotypes of bacterial communities were annotated, including aerobic, anaerobic, contains_mobile_elements, forms_biofilms, facultatively_anaerobic, gram_negative, gram_positive, potentially_pathogenic, and stress_tolerant. The phyla Proteobacteria and the genera *Acinetobacter*, *Cupriavidus*, and *Pseudomonas* were the stress_tolerant and potentially_pathogenic bacteria. Lungs of neonatal pigs in the ATRA4, ATRA16, and ATRA32 groups had a lower (*p* < 0.05) percentage of stress_tolerant and potentially_pathogenic bacteria than that of neonatal pigs in the ATRA0 group, respectively. The phyla Acitobacteria and Firmicutes and the genera *Clostridium* and *Streptococcus* were the contributors of gram_positive bateria, and neonatal pigs in the ATRA32 group had a higher (*p* < 0.05) percentage of gram_positive bacteria in lungs compared to neonatal pigs in the ATRA0 group. The annotated gram_negative bacteria were the phyla Proteobacteria and Cynobacteria and the genera *Acinetobacter*, *Cupriavidus*, *Pseudomonas*, *Nelumbo*, *Sphingobium*, and *Sphingomonas*, and neonatal pigs in the ATRA32 group had a lower (*p* < 0.05) percentage of gram_negative bacteria in the lungs compared to neonatal pigs in the ATRA0 group.

### 3.7. Differentially Expressed Genes, Kyoto Encyclopedia of Genes and Genomes (KEGG) Pathway Enrichment

**Figure 12:**
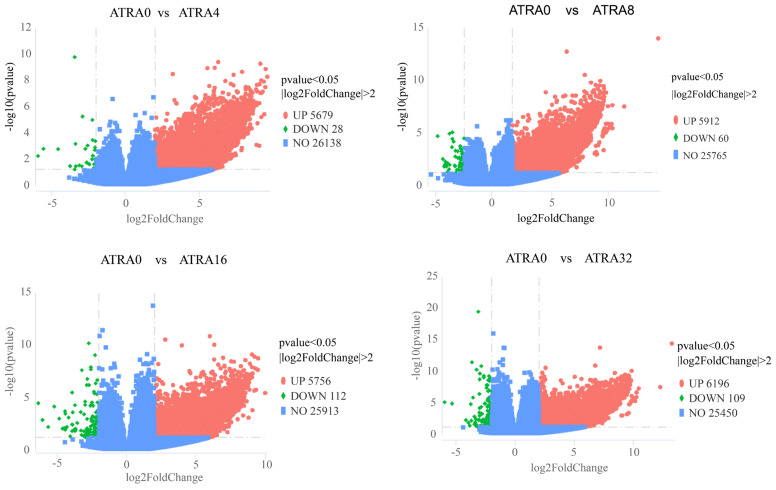
Volcano plot of differentially expressed genes in lungs between groups (*n* = 6 per group). ATRA0: supplementation of ATRA at 0 mg/kg basal diet; ATRA4: supplementation of ATRA at 4 mg/kg basal diet; ATRA8: supplementation of ATRA at 8 mg/kg basal diet; ATRA16: supplementation of ATRA at 16 mg/kg basal diet; ATRA32: supplementation of ATRA at 32 mg/kg basal diet. The abscissa represents the fold changes in gene expression. The ordinate represents the statistical significance of the variations in gene expression.

**Figure 13:**
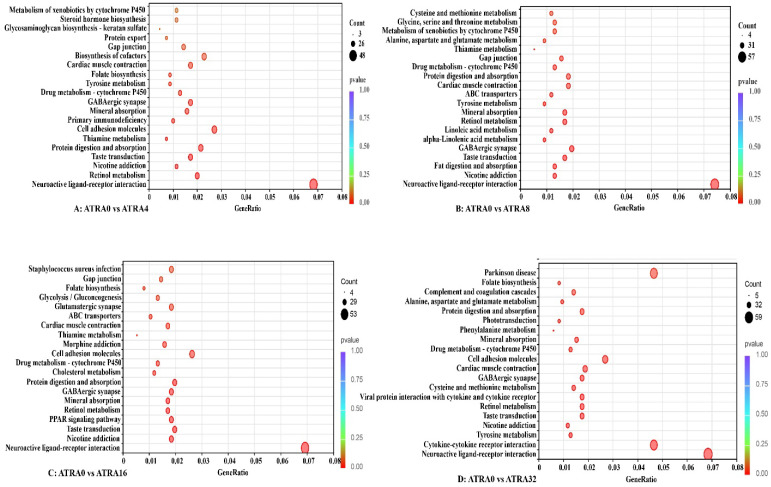
KEGG pathways of differentially expressed genes in lungs between groups (*n* = 6 per group). ATRA0: supplementation of ATRA at 0 mg/kg basal diet; ATRA4: supplementation of ATRA at 4 mg/kg basal diet; ATRA8: supplementation of ATRA at 8 mg/kg basal diet; ATRA16: supplementation of ATRA at 16 mg/kg basal diet; ATRA32: supplementation of ATRA at 32 mg/kg basal diet.

Figure 12 shows that the number of differentially expressed genes was 5707 (5679 up, 28 down) between the ATRA0 group and ATRA4 group, 5972 (5912 up, 60 down) between the ATRA0 group and ATRA8 group, 5868 (5756 up, 112 down) between the ATRA0 group and ATRA16, and 6305 (6196 up, 109 down) between the ATRA0 group and ATRA32 group, respectively. The differentially expressed genes between groups were blasted to the KEGG database to analyze their biological pathways, and the top 20 abundant pathways between groups are presented in Figure 13. The common KEGG pathways shared by the differentially expressed genes in the four comparison groups were drug metabolism–cytochrome P450, GABAergic synapse, mineral absorption, protein digestion and absorption, nicotine addiction, retinol metabolism, and neuroactive ligand–receptor interaction. The results of KEGG pathway enrichment indicated that supplementation of ATRA to pregnant sows altered the expression of genes in lung tissue of neonatal pigs and increased the number of genes related to neural development. The genes shared by the four comparison groups in each KEGG pathway are listed in Table 2.

## 4. Discussion

The normal development of the fetal lungs is crucial for postnatal respiratory health and production in animals, and nutrition can modulate the inflammatory status, morphology, gene expression, and microbial composition of the lungs [4,31,32,33,34]. Previous studies indicated that when mice suffered from protein restriction during pregnancy, it decreased the relative mass of the lungs in neonates (*p* < 0.05), lowering the levels of TNF-α (*p* > 0.05), IL-6 (*p* > 0.05), and IL-10 (*p* < 0.05) [35]. Hoxa1−/−neonatal pigs delivered by crossbred sows (Erhualian × Shaziling) supplemented with ATRA at 4 mg/kg bodyweight on dpc 14 had higher concentrations of INF-γ (*p* < 0.05), TNF-α (*p* > 0.05), and IL-8 (*p* > 0.05) in lung tissue compared to Hoxa1^−/−^ neonatal pigs born from hybrid sows (Erhualian × Shaziling) without ATRA [4]. The experimental results demonstrated that the use of IL-6 in late pregnancy may be beneficial in the latter stages of fetal lung maturation [36]. IL-10 is an anti-inflammatory cytokine, and in a steady state, it can maintain commensal balance and immune homeostasis in the lungs [37,38]. TNF-α at an optimal level plays crucial roles in pathogen clearance and newborn health protection [39]. Excessive or insufficient levels of these cytokines can damage the normal development of fetal lungs, characterized by symptoms of dyspnea and abdominal respiration [4], and low levels of TNF-α, IL-6, and IL-10 can result in the simplification of the alveoli [35]; the results of this study indicated that supplementation of ATRA at 4 mg/kg diet in pregnant sows can positively improve the histomorphological structure of lungs in neonatal pigs through a moderately high level of IL-1β, IL-6, IL-10, IL-13, and TNF-α in lung tissues.

AQP5 and SFTPC play pivotal roles in maintaining the normal morphology and function of alveoli. AQP5 can be expressed in Type 1 alveolar epithelial (AT1) cells and functions in controlling membrane permeability [40]. SFTPC is distributed in Type 2 alveolar epithelial (AT2) cells and has the ability to reduce alveolar surface tension and increase membrane permeability [41]; excessive SFTPC expression can cause edema by forcing large amounts of alveolar fluid into lung tissues [42,43]. ATRA can induce AT2 differentiation to AT1 and increase AQP5 expression [44]. Chen et al. (2023) reported that Hoxa1 mutation disturbed the balance between AQP5 and SFTPC, and the administration of ATRA to pregnant sows improved the balance between AQP5 expression and SFTPC expression by increasing AQP5 expression and decreasing SFTPC expression [4]. The results of this study further verified this effect of maternal administration with ATRA at 4 mg/kg diet.

The fetal lung was usually considered a sterile organ, but now, it is well demonstrated that this organ harbors a variety of microorganisms [38]. Previous studies indicated that early interventions in maternal nutrition and contact had significant influences on the maturation, immunity, and microbial composition of the lungs in neonatal piglets [45,46]. Lots of experiments reported that the microbiota in the fetal lungs is dominated by Bacteroidetes, Firmicutes, Proteobacteria, Actinobacteria, Fusobacteria, and Saccharibacteria at the phylum level [47,48] and by *Prevotella*, *Streptococcus*, *Veillonella*, *Pseudomonas*, *Fusobacteria*, *Haemophilus*, and *Neisseria* at the genus level [49,50]. However, this study showed that the bacterial composition in the lungs of neonatal pigs from five ATRA treatment groups was dominated by Firmicutes, Proteobacteria, Actinobacteriota, Cyanobacteria, and Bacteroidota at the phylum level and by *Clostridium_sensu_stricto_1*, *Actinetobacter*, *Streptococcus*, and *Escherichia-Shigella* at the genus level, respectively.

Proteobacteria, including the genera of *Haemophilus*, *Neisseria*, *Pseudomonas*, *Rickettsia*, and *Moraxella*, have been identified in asthma and are usually associated with uncontrolled asthma [51]. In lots of chronic lung diseases, there is an outgrowth of proteobacteria like *Pseudomonas aeruginosa* [52,53], and *Pseudomonas aeruginosa* has been identified in the lungs of patients with asthma [54]. *Acinetobacter* is an opportunistic pathogen, which can result in pneumonia [55]; it can also induce lung cell death by increasing oxidative stress and cytosolic calcium release [56]. The relative abundance of *Acinetobacter* is significantly elevated in the lungs of patients with chronic obstructive pulmonary disease (COPD) [57]. *Stenotrophomonas* is considered as one of the drug-resistant pathogens, and it can exacerbate lung infection [58]. As a species of *Enterobacter*, *Enterobacter hormaechei* was reported to damage lung tissue in unweaned calves with a thickened alveoli septum and inflammatory cell infiltration, and this pathogen has resistance to β-lactam antimicrobials [59]. *Alistipes* is positively associated with chronic inflammation [60]. *Akkermansia* can protect animals from infection by pathogens, block the virulence factor expression of Fusobacterium nucleatum, increase anti-inflammatory responses mediated by IL-10 and expression of tight junction proteins that limit pathogen translocation across epithelial barriers [61,62]. The LEfSe results of this study indicated that neonatal pigs in the ATRA4 group had a lower (*p* < 0.05) relative abundance of Proteobacteria, *Acinetobacter*, *Cutibacterium*, *Stenotrophomonas*, *Enterobacter*, *Alistipes*, and *Saccharomonospora* and a higher (*p* < 0.05) relative abundance of *Gemmatimonadota*, *unidentified_Mitochondria*, and *Akkermansia* in the lungs than neonatal pigs in ATRA0, respectively. It implies that the maternal administration of ATRA at 4 mg/kg diet contributes to reducing lung diseases and antibiotic resistance in newborn piglets.

Bacteria have substantial functions in regulating organ health in animals. Previous studies showed that COG2197 is related to bacterial virulence [63], COG1280 is essential for bacterial virulence factor production, biofilm formation, and antibiotic resistance [64,65], COG0845 and COG1113 can increase drug resistance [66,67], COG0768 has a role in resistance to β-lactam antibiotics [68], and COG0735 can inhibit siderophore synthesis in pathogens and increase virulence of pathogens [69]. Data on COG function prediction showed that supplementation of ATRA at 4 mg/kg diet in pregnant sows significantly decreased the relative abundances of COG2197, COG1280, COG0845, and COG1113 but increased the relative abundances of COG0768 and COG0735 in lung bacteria in neonatal pigs. It hints that maternal supplementation with ATRA at 4 mg/kg diet has the benefits in reducing pathogen virulence and drug resistance but has the risk of increasing resistance to β-lactam antibiotics. Predicting microbial phenotypes is an alternative method to understand the classification, pathogenicity, adaptability, and drug sensitivity of the microbiota in animal tissues, providing new insights into the prevention and treatment of diseases. Data from bacterial BugBase prediction indicated that the supplementation of ATRA at 4 mg/kg diet to pregnant sows from dpc 12 to 95 effectively decreased the relative abundance of potential pathogens (Proteobacteria, *Acinetobacter*, *Cupriavidus*, and *Pseudomonas*) and alleviated the stress tolerance capacity of these potential pathogens in the lungs of neonatal pigs.

Genes also play critical roles in the health status of animals. Previous findings validated that UTS2R is a G protein-coupled receptor, and the activation of UTS2R in neurons can influence its neurotransmission [70]. RSAD2 participates in differentiation of macrophages and T cells and can protect animals from viral infections [71,72]. RSAD2 excess increases lipid droplet accumulation and interferon levels [73,74]. GBP1 can promote cell-intrinsic defense by attacking intracellular pathogens via autophagy, oxidative responses, inflammasomes, and cell death and by inducing programmed cell death of pathogens such as *Chlamydia* and *Staphylococcus* [75,76]. ANGPTL3 is involved in new blood vessel growth [77]. THBS1 promotes angiogenesis by the HIF-1/VEGF signaling pathway [78], and it is also associated with cell proliferation and apoptosis; a decrease in THBS1 expression can accelerate cell apoptosis, and an increased rate of cell apoptosis is also one of the causes of pulmonary fibrosis [79]. Decreasing SPOCD1 expression can attenuate extracellular matrix deposition [80]; it might be one of the factors for the decreased thickness of alveolar septum. ABCC12 is one of the multidrug resistance proteins and participates in the intracellular drug efflux process, leading to multidrug resistance [81]. SLITRK1 is associated with neurite development and synaptogenesis [82], and increased CCL11 expression can exacerbate asthma [83]. Nutrition influences animal health by regulating gene expression in organs; the data of this study demonstrated that supplementation of ATRA in pregnant sows at 4 mg/kg diet on dpc 12 significantly increased the expression of UTS2R, RSAD2, TREML1, GBP1, CD4, ANGPTL3, TRNAV-AAC, TNFSF15, THBS1, and RGS9BP and decreased the expression of SPOCD1, CBLN4, BARHL2, SLITRK1, SERPINB12, TAS2R41, ABCC12, CALB2, ACTL9, TRNAP-AGG, and CCL11. This indicates that maternal administration with ATRA at 4 mg/kg diet can strengthen the health and function of the lungs in neonatal pigs by activating neurotransmission, attacking pathogens, alleviating drug resistance and asthma, and decreasing alveolar septum thickness through regulating gene expression in the lungs.

## 5. Conclusions

Maternal supplementation of ATRA at 4 mg/kg diet from dpc 12 to 95 has no significant influences on the levels of pro-inflammatory and anti-inflammatory cytokines compared to ATRA addition at 0 mg/kg diet. But importantly, feeding sows with the diet supplemented with ATRA at 4 mg/kg diet from dpc 12 to 95 can strengthen the development and health of the fetal lungs by increasing the number of alveoli, decreasing the thickness of the alveolar septum, reducing the number, virulence, drug resistance, and stress tolerance capacity of pulmonary pathogens, enriching the highest number of differentially expressed genes in the pathway of neuroactive ligand–receptor interaction, and down-regulating the expression of asthma-related genes. This practice could be beneficial in increasing survival rate and growth performance by reducing lung diseases in piglets.

## Figures and Tables

**Table 1:** Level of cytokines in lungs of neonatal pigs from different ATRA groups.

	ATRA0 (*n* = 6)	ATRA4 (*n* = 6)	ATRA8 (*n* = 6)	ATRA16 (*n* = 6)	ATRA32 (*n* = 6)
IL-1β (pg/g)	89.66 ± 2.28 ^ab^	98.30 ± 4.35 ^ab^	87.63 ± 3.45 ^ab^	103.76 ± 1.98 ^a^	81.91 ± 8.01 ^b^
IL-6 (pg/g)	106.56 ± 3.36 ^B^	118.02 ± 3.71 ^AB^	95.20 ± 2.43 ^C^	130.61 ± 4.41 ^A^	97.73 ± 2.75 ^C^
IL-10 (pg/g)	113.25 ± 1.85 ^ab^	123.43 ± 4.13 ^ab^	115.46 ± 3.58 ^ab^	126.02 ± 5.36 ^a^	109.03 ± 1.83 ^b^
IL-13 (pg/g)	106.34 ± 2.09 ^ABab^	112.06 ± 4.59 ^ABa^	95.90 ± 2.48 ^Bb^	117.45 ± 4.79 ^Aa^	94.17 ± 3.16 ^Bb^
TNF-α (pg/g)	110.29 ± 3.07 ^ABab^	120.10 ± 2.44 ^ABa^	105.36 ± 3.70 ^Bb^	118.26 ± 4.52 ^ABa^	102.52 ± 2.21 ^Bb^

ATRA0: Supplementation of ATRA at 0 mg/kg basal diet; ATRA4: supplementation of ATRA at 4 mg/kg basal diet; ATRA8: supplementation of ATRA at 8 mg/kg basal diet; ATRA16: supplementation of ATRA at 16 mg/kg basal diet; ATRA32: supplementation of ATRA at 32 mg/kg basal diet. Data are presented as “Mean ± SEM”. Mean values within a row differ significantly at *p* < 0.01 with different capital letters or at *p* < 0.05 with different lowercase letters.

**Table 2:** Information on KEGG pathways and genes shared by four comparison groups (*n* = 6 per group).

KEGG Pathway	Genes Shared by Four Groups in Same Pathways
Neuroactive ligand–receptor interaction	UTS2R, NPY, ADORA2A, GALR3, TACR2, MLNR, GHRL, UCN, GRPR, GLRA1, TACR1, KISS1, DRD2, INSL3, GRM5, PNOC, RXFP1, CRSP3, TAC4, GRIK1, GALR2, NPFFR1, AVP, GLP1R, TRPV1, CHRNA6, GABRR1, GABRR2, GABRA5, GABRA6, GABRB3, GABRQ
GABAergic synapse	SLC6A1, SLC6A13, HAP1, GAD1, GAD2, GABRR1, GABRR2, GABRB3, GABRA5, GABRA6, GABRQ
Nicotine addiction	GABRR1, GABRR2, GABRA5, GABRA6, GABRB3, GABRQ, CHRNA6
Drug metabolism–cytochrome P450	AOX2, AOX4
Retinol metabolism	UGT1A6, RDH12, AOX2, LRAT, RPE65, AOX4
Mineral absorption	ATP1A3, SLC39A4, SLC34A1
Protein digestion and absorption	KCNN4, ATP1A3, MEP1A, SLC15A1, MEP1B, COL22A1, PGA5, CPA2, CPA3, SLC36A3
Asthma	CCL11, IL9

## Data Availability

The data presented in this study are openly available at [Sequence Read Archive of the National Center for Biotechnology Information] at [https://www.ncbi.nlm.nih.gov/sra/?term=PRJNA1233939, accessed on 10 March 2025].
